# Assessment of motivational interviewing: a qualitative study of response process validity, content validity and feasibility of the motivational interviewing target scheme (MITS) in general practice

**DOI:** 10.1186/s12909-017-1052-7

**Published:** 2017-11-21

**Authors:** Riëtta Oberink, Saskia M. Boom, Nynke van Dijk, Mechteld R. M. Visser

**Affiliations:** Department of General Practice/Family Medicine, Academic Medical Centre–University of Amsterdam, meibergdreef 15, 1105 Amsterdam, The Netherlands

**Keywords:** Motivational interviewing, Assessment, Education, General practice

## Abstract

**Background:**

The Motivational Interviewing target Scheme (MITS) is an instrument to assess competency in Motivational Interviewing (MI) and can be used to assess MI in long and brief consultations. In this qualitative study we examined two sources of the Unified Model of Validity, the current standard of assessment validation, in the context of General Practice. We collected evidence concerning response process validity and content validity of the MITS in general practice. Furthermore, we investigated its feasibility.

**Methods:**

Assessing consultations of General Practitioners and GP-trainees (GPs), the assessors systematically noted down their considerations concerning the scoring process and the content of the MITS in a semi-structured questionnaire. Sampling of the GPs was based on maximum variation and data saturation was used as a stopping criterion. An inductive approach was used to analyse the data. In response to scoring problems the score options were adjusted and all consultations were assessed using the original and the adjusted score options.

**Results:**

Twenty seven assessments were needed to reach data saturation. In most consultations, the health behaviour was not the reason for encounter but was discussed on top of discussing physical problems. The topic that was most discussed in the consultations was smoking cigarettes. The adjusted score options increased the response process validity; they were more in agreement with theoretical constructs and the observed quality of MI in the consultations. Concerning content validity, we found that the MITS represents the broad spectrum and the current understanding of MI. Furthermore, the MITS proved to be feasible to assess MI in brief consultations in general practice.

**Conclusions:**

Based on the collected evidence the MITS seems a promising instrument to measure MI interviewing in brief consultations. The evidence gathered in this study lays the foundation for research into other aspects of validation.

**Electronic supplementary material:**

The online version of this article (doi: 10.1186/s12909-017-1052-7) contains supplementary material, which is available to authorized users.

## Background

“Motivational Interviewing (MI) is a collaborative, goal oriented style of communication with particular attention to the language of change. It is designed to strengthen personal motivation for and commitment to a specific goal by eliciting and exploring the person’s own reasons for change within an atmosphere of acceptance and compassion” [1]. MI is widely disseminated in mental and medical care settings [1–3]. It was found efficacious in primary care when used alongside or within the delivery of routine medical care to support the treatment of chronic diseases and for preventive purposes [3].

In the Netherlands, GPs see their patients on average four times a year [4] which provides an opportunity to invite patients to discuss behaviour change. Since the role of Dutch GPs is increasingly focused on prevention of diseases, interventions that facilitate behaviour change are incorporated into the curriculum of GP-trainees. In 2012 MI became a required component of the Dutch curriculum [5]. Although MI may seem quite simple to apply, the practice turns out to be less easy. Practitioners might think they apply MI while they actually do not, and even use MI-inconsistent behaviour (e.g., giving advice without permission, confronting, setting goals for patients) which is considered to be related to a poor outcome in terms of behaviour change [6–9]. Therefore, thorough training in MI including assessment of MI fidelity and the quality of the skills is needed [10].

Validated instruments are needed to assess the quality of MI. To assess MI skills in GP residents, an instrument is required that is feasible in the GP-setting and from which valid conclusions can be derived. Widely used instruments like the Motivational Interviewing Skill Code (MISC) [11] and the Motivational Interviewing Treatment Integrity scale 3.1.1 (MITI) [12] are less suitable because they require practice samples that are longer than those usually available in general practice, in which consultations generally last 10–15 min. The Behaviour Change Counselling Index (BECCI) [13] was developed to help trainers evaluate MI-skills acquired in training but was not constructed to measure MI in real consultations.

The Motivational Interviewing Target Scheme (MITS) [14] was developed to assess global core components of MI, also in brief conversations. In global assessment, skills are grouped in one or more overarching constructs and are not assessed as isolated skills or steps as in checklists. This is thought to give a better reflection of competence, especially when complex skills, as in MI, are measured [15, 16] The MITS can be used to give feedback (formative assessment), for selection (summative assessment) and for research purposes [14].

The MITS has been thoroughly constructed. The developers strived for incorporating all the essential behaviours of MI in global items. They considered descriptions provided by the founders of MI and MI-assessment instruments. This resulted in definitions that described the core constructs of MI behaviour. Subsequently they tested if the behaviour could be observed in practice samples and received feedback from MI-experts. If necessary, the core constructs were adjusted after consensus discussions [14].

This method of developing the instrument contributed to the validity of the MITS as described in the Unified Model of Validity, the current standard for assessment validation [17, 18]. In this model validity is not a property of the instrument as such, but refers to the extent to which interpretations of the scores of an instrument are valid for the population under scrutiny. Moreover, every step, from the development of the instrument until the interpretation of the scores in the target population, should be studies to provide a complete picture of the validity. Validity evidence is collected from five sources (see Table 1) and should be assembled in a structured way [18, 19]. In contrast to earlier models of assessment validation, qualitative research plays an important part in the Unified Model of Validation and precedes quantitative methods [20].

**Table 1:** Unified Model of Validity: The five sources of validity to support construct validity^a^

Source	
Content	Refers to themes, wording, and format of items, and includes expert review and other systematic item development strategies.
Response Process	Is about the actions and thought processes of the test-users (including scoring) that reduce the likelihood of response error.
Internal Structure	Concerns acceptable reliability and factor structure.
Relation to Other Variables	Concerns correlation with scores from other instruments.
Consequences	Is about the soundness of test score based decisions.

^a^ In the Unified Model of Validity, all validity should be conceptualized under one overarching framework, the “construct validity” [17]

In the present qualitative study we investigated several aspects of the feasibility of the MITS in general practice and collected evidence concerning content and response process validity. Concerning content validity, we examined the extent to which the items of the MITS cover the full spectrum of MI. According to Cook et al. [17] response processes are rarely examined but it is important to do so to understand and reduce response error. Kreiter [20] stresses the importance of response process validity in achievement testing as it is concerned with establishing whether the items are likely to require the behavioural skill we seek to assess. The evidence may be derived from asking the test users what strategies they use to respond or by analysing the steps required producing the ‘correct’ answer.

We collected response process evidence by analysing the test users’ (in this study: the assessors) considerations during the assessment process. This provided information on how well assessors’ responses align with the intended construct.

## Methods

### Setting and participants

To become a GP in the Netherlands, registered medical doctors enrol in a 3-year program combining education at one of the eight institutes (1 day a week) and work in a training practice under supervision of a GP. We conducted this study at the GP Specialty Training Program at the Academic Medical Centre (AMC) of the University of Amsterdam. In year three of the GP-training, MI forms part of the doctor-patient communication training (three 3-h sessions in groups of 6–8, spread over 6 months). Under supervision of a psychologist who is experienced in MI, trainees discuss consultations they recorded in practice. Assessment of consultations in which MI is used is part of their exam.

We selected 11 GPs and GP-trainees^1^ who had handed in 2–3 consultations on behaviour change; nine GP-trainees and two GPs working at the department of GP training at the AMC (May 2013). Participation was voluntary. Participants received written information and were asked to give written informed consent. Informed consent of the patients was recorded. The study is approved by the Ethical Review Board of the Dutch Association for Medical Education (NVMO NEBR 222) and was exempt from medical ethical review.

### Design

Maximum variation sampling was used to obtain a diverse group of respondents. GPs were selected based on age, gender, nationality and former experience as these aspects are supposed to be related to the quality of communication [21]. From every GP one consultation was randomly selected from 2 to 3 consultations and was subsequently scored with the MITS by three assessors who are familiar with MI and with the GP-setting.

Data on validity evidence and feasibility were gathered by way of a semi-structured questionnaire and by notes made during evaluation meetings of the assessors that took place after every 2–3 assessments. The assessors filled out the semi-structured questionnaires during the scoring process. All data were coded and categorized. Data saturation was used as a stopping criterion and was reached when no new codes or categories occurred.

During the first evaluation meeting the assessors discussed problems they came across while using the score options. In response to these scoring problems in the first part of the study (the first phase), the score options were adjusted. Subsequently, all consultations were rated both with the original and the adjusted score options (second phase).

### Motivational interviewing target scheme (MITS)

**Fig. 1:**
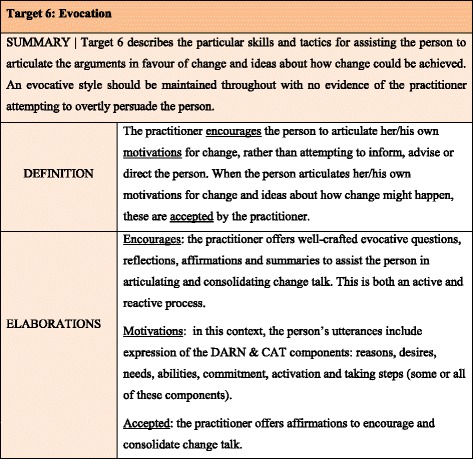
Example of a Target of the MITS (Printed with permission of J. Allison)

In the MITS [14], MI-consistent practice is described in 10 global items (targets) (Table 2). Every target has a summary, a definition and elaborations (Fig. 1). The score options (0–4) represent the degree to which the target definitions are met (Table 3). Targets 1–7 are regarded as necessary components of MI and should be scored. Targets 8–10 are only scored when applicable.

**Table 2:** The 10 targets of the MITS

Target	Summary (for a complete description see the manual of the MITS)
1. Activity Emphasis	Target 1 describes a flexible framework of three primary activities (Considering, Discussing and Advocating) within which the other target behaviours may be practised. The practitioner uses the activity that will best serve the general strategic goal of increasing the likelihood of movement towards change.
2 Posture	Target 2 describes the preferred manner in which the practitioner should conduct her/himself at all times. This posture is consistent with enhancing effectiveness, common decency and, above all, doing no harm.
3 Empathy	Target 3 describes the core skills of discriminating empathic reflection. Its employment has many purposes in MI including, achieving and maintaining harmonious relations (rapprochement), steering the course of the conversation, and building the case for change.
4. Collaboration	Target 4 describes the sense of purposeful collaboration, of which all parties to the conversation become aware.
5. Independence	Target 5 describes a foundation aspect of the relationship, in which the practitioner works to establish, legitimise and maintain recognition of the person’s independence with regard to all matters pertaining to the focal predicament.
6. Evocation	Target 6 describes the particular skills and tactics for assisting the person to articulate the arguments in favour of change and ideas about how change could be achieved. An evocative style should be maintained throughout with no evidence of the practitioner attempting to overtly persuade the person.
7. Navigation	Target 7 describes the skills of ‘pushing forward’ the conversation in a promising and ultimately productive direction, taking the person along as a willing collaborator, and without causing things to fall apart - disengagement.
8. Contrasts	Target 8 describes the skills of causing the person to consider apparent inconsistencies between the conversation’s focal problem and her/his goals, aspirations, beliefs or values, without evoking a sense of despondency or hopelessness.
9. Structured brief tactics	Target 9 is concerned with the skills of employing, intermittently, particular conversational ‘set routines’ that assist in clarifying the goals of change, the state of readiness, factors inhibiting and facilitating change, or the route toward declared goals etc.
10. Information & Advice	Target 10 is concerned with the skills of giving information and advice with effect; that is, in such a manner that the person most likely will at least consider it, it if not act upon it.

**Table 3:** Target Score Options of the original MITS

Score option	Description
0	There is no evidence or almost no evidence to support the target definition Definition no evidence: in no part; to no degree. Synonyms: not any, nothing, not a bit, not a hint.
1	The evidence partly supports the target definition Definition partly: in part; to some degree. Synonyms: after a fashion, appreciably, at the least, in a manner, in a way, in small part, in some measure, incompletely, partially.
2	The evidence moderately supports the target definition Definition moderately: to some extent; to a mainly sufficient degree*.* Synonyms: reasonably well, somewhat, middling, passably, acceptably, nominally, somewhat, tolerably.
3	The evidence substantially supports the target definition Definition substantially: to a great extent; to a large degree. Synonyms: considerably, more than adequately, amply, largely, mainly, mostly, on the whole.
4	The evidence completely or almost completely supports the target definition Definition completely: to the highest degree; with everything necessary. Synonyms: to the full or entire extent, wholly, entirely, totally, all, altogether, comprehensively, exhaustively. Definition almost: for the most part; very nearly all

The assessment procedure is described as follows: a sample set should be composed of recordings of complete individual MI sessions (with a total of 30–45 min, containing 1–6 samples) and preferably accompanied with a transcript. The assessor views and/or listens to all recordings and reads the transcripts in its entirety. Subsequently, the score options that best describe the holistic impression of the observed behaviour will be selected for every target. The mean score of the targets determines the final score ranging from 0 (*no MI-consistency*) till 4 (*very high degree of MI consistency*).

For this qualitative study we assessed one consultation at a time to investigate the content of the targets and the thought processes of the assessors. This approach is consistent with the use of communication rating scales in which the final score is the mean of all assessed consultations [16, 22, 23].

### Translation of the MITS and training

The translation of the MITS followed a forward–backward procedure. Two psychologists translated the MITS independently and then discussed what best reflected the descriptions in the MITS. A native speaker translated the result back into English. The backward translation was compared with the original text, and adjustments were made to the Dutch translation if necessary. All translators were familiar with MI-terminology.

Three psychologists, familiar with MI, were trained to use the MITS by one of the developers (RB) during three 3-h sessions spread over 3 months. During the training the scores of three consultations were discussed until consensus was reached on the interpretation of the targets and the rating. After this training, the assessors continued to discuss their ratings during the evaluation meetings in order to maintain or improve the inter-rater agreement.

### Data collection

We requested participants to hand in 2–3 video recordings of consultations with patients on behaviour change. It was not required that the participants consciously applied MI. The recordings were stored on a secure server at the AMC.

Three assessors assessed one consultation of each participant, seven with and four without a transcript. The consultations were viewed once and it was allowed to pause the recording to take notes. If the assessors were not sure about their initial rating, they could watch the video a second time. If they wanted to change their initial ratings, they added new ratings, keeping the initial ratings on record. During this process they filled out a semi-structured questionnaire (see Table 4). After every 2–3 assessments, evaluation meetings took place to discuss and note down scoring problems, resulting in adjusted score options. All consultations were subsequently rated both with the original and the adjusted score options.

**Table 4:** Semi-structured questionnaire

Questions	Rating
1. First phase of the study: Rate each target of the MITS with the original coding system. Second phase of the study: Rate each target of the MITS with the original and additional coding system ^(1)^. Any comments?	0–4 0–4 Open answer
2. What problems did you face? / What made it easy to assess? Why?	Open answer
3. Did you watch once or twice?	
4. Is this consultation useful for this study? Why?	Yes, no Open answer
5. What was the reason for consulting the GP?	Open answer
6. What behaviour change is discussed?	Open answer
7. What was the duration of the consultation? (Without physical examination).	Minutes
8. How much time is spent on MI?	Minutes
9. How much time did it take to assess?	Minutes

^1^This item was added on the basis of the scoring problems detected during the first phase

For the Dutch version that was used in this study see Additional file 1: Semi-gestructureerde vragenlijst beoordeling MITS

### Instruments

#### Questionnaire on background information

The participants provided biographical information (sex, age, GP or GP-trainee) and information on familiarity with MI (Did you read about MI? What did you read? Are you trained in MI? How many hours?)

#### Semi-structured questionnaire

The assessors answered questions concerning the characteristics of the consultation, the feasibility of the MITS to assess brief consultations and its usefulness in the GP setting (Table 4). They noted down comments per target and on the assessment as a whole.

#### Analysis

An inductive approach was used. Two authors (RO and MV) categorized the comments made by the assessors independently. After a consensus discussion the categories were slightly adapted. A descriptive analysis of the ratings of the MITS was made. Microsoft Excel (14.1.0) was used to organize the data.

## Results

### Characteristic of the participants and consultations

After 27 assessments (9 out of 11 consultations rated by three assessors) data saturation was reached. Table 5 shows the characteristics of the GPs and the consultations.

**Table 5:** Characteristics of the participants and their consultation

Characteristic	Mean (SD)	%/ Range
Participants (*n* = 9)		
GPs age (*n* = 2)	56.5 (0,7)	
GP-trainee age (*n* = 7)	34.4 (3.3)	
Dutch		78
Female		67
Read about MI before		100
Did not receive any training in MI		11
More than four hours training in MI		44
Consultations		
Reason for encounter: physical problem		78
Smoking cigarettes was discussed		78
Physical exercise and diet were discussed		11
Smoking cigarettes and losing weight were discussed		11
Duration of consultation (minutes)	14 (3.4)	(Range 11–20)
Time spent on discussing behaviour change (minutes)	7.3 (3.2)	(Range 2.3–10.3)

### The first phase of the study

The assessors encountered the following problems concerning the score options during the first phase of the study.

### Problems related to the score options

According to the assessors the adverbs used to describe one score option (Table 3) did not always have the same meaning. A quote on score option “3” was:

*“The score matches ‘substantially’ but not ‘more than adequately’ ”. (Assessor 2)*

Furthermore, the assessors had different views on the meaning of some descriptions (which are a mixture of quantitative and qualitative adverbs) and the same findings resulted in different ratings. Therefore they looked for less ambiguous adverbs. This resulted in four qualitative adverbs (poorly, inadequately, moderately, well), which were included in the adjusted score options (see Table 6). The extent to which the quantity must be taken into account is described per score option (e.g. Score option 3: “The target behaviour is well implemented during the *majority* of the time”).

**Table 6:** The adjusted score options

Score option	Description
0	The target behaviour is poorly implemented most of the time or wrongly unused.
1	The target behaviour is inadequately implemented. Possibly there is target-inconsistent behaviour and / or multiple missed opportunities.
2	The target behaviour is reasonably implemented. There is no target-inconsistent behaviour and / or limited missed opportunities. Or The target behaviour is well implemented during the majority of the time and only occasionally there is target-inconsistent behaviour and / or a limited number of missed opportunities.
3	The target behaviour is well implemented. There is no target inconsistent behaviour. There is a limited number of missed opportunities
4	The target behaviour is well implemented. There is no target inconsistent behaviour. There are no important missed opportunities

### Problems related to the scoring of the target behaviour

- The target behaviour is wrongly unused
  According to the MITS manual target 8–10 should only be coded when the behaviour is observed and cannot get the zero value. However, the assessors noticed that there is a distinction between target behaviour that was ‘rightly unused’ and target behaviour that was ‘wrongly unused’. An illustration of this from one of the consultations is:
*When a patient asked for information to quit smoking, the GP started to ask about the pros and cons of smoking and during that consultation no information was given at all.*

According to the original score options this would be coded as ‘not applicable’ at target 10. In the adjusted score options we added the possibility to score a zero when the target behaviour was ‘wrongly unused’ (Table 6) and the omission was crucial and therefore not in line with good MI-practice.

b. Missed opportunities within the target behaviour
  Close to the former theme are ‘missed opportunities’. The difference is that the practitioner does not fail to use the target behaviour but within the target behaviour obvious chances are unused. We added the possibility to score ‘missed opportunities in the adjusted score options. A reactions on this adaptation is:
*“The score is more appropriate now because I can rate the ‘quality of what was done’, but also the impact of ‘what is not done’ ”. (Assessor 3)*

c. MI / target-inconsistent behaviour
  MI-inconsistent behaviour is not mentioned in the original score options. This caused problems when the target behaviour was performed well during the majority of the time but some MI/target-inconsistent behaviour was shown as well. It was not clear what the impact of the MI/target-inconsistent behaviour should be on the scoring. To solve this problem, we added ‘target-inconsistent behaviour’ to the score options (See Table 6).
  A quote on this adjustment was:
*“It is clear that the score cannot exceed a ‘2’ because of target-inconsistent behaviour”. (Assessor 1).*

### The second phase of the study

In the second phase of the study, all consultations were scored with the original and the new score options. The semi-structured questionnaires (Table 4) yielded 315 comments, which were coded and categorized. Three categories emerged, consisting of comments on: the score options, assessment in general, and the targets (Table 7). These categories provide information on feasibility and validity evidence in the GP-setting and will be described below as well as the distribution of the ratings over the five score options.

**Table 7:** Categories resulting from the answers on the semi-structured questionnaire

1. Comments related to Response Process evidence: Comments on the score options	2. Comments related to Response Process: Assessment problems in general	3. Comments related to Content evidence
The score option does not provide sufficient guidance	Other parts of the consultation (not on behaviour change) have influenced the assessment	The description of the target is not clear (e.g. target 3)
How to score missed opportunities	Difficult to give very low or high marks	The description is expanded and/or complicated
How to score MI-inconsistent behaviour	Follow-up consultation (it is not clear what preceded)	Targets are overlapping (e.g. target 2 and 4)
How to score the various components in a target when some occur and others not or badly	It is easier to assess good consultations	It is not clear if the content or the process of the target should be rated or both (e.g. target 4)
How to score when the target behaviour is well done in (most) parts of the consultation and not or poorly in other parts		Target inconsistent behaviour is not described in the target (e.g. target 6)
		The step to the planning phase or follow-up is not mentioned explicitly

### Feasibility in GP setting

- Time spent on the assessment by the assessors and the use of transcripts
  During the first assessments it took the assessors 75–120 min to watch the whole consultation, to code the MI-part and answer the questionnaire (which is normally not part of the scoring procedure). They preferred to watch (parts of) the consultations twice but after 5 assessments, they did this only occasionally. After 5 assessments, if the assessors used a transcript of the consultation and took notes, it took about 30 min to assess 10 min of MI. Without transcript the assessment total coding time increased by approximately 10–15 min as recordings were paused more often and more notes were written down.

b. Assessing short fragments spent on behaviour change
  Two assessors found it difficult to give a global rating to target 3 (empathy) when the part of the consultation on behaviour change was short (2.3 min), the third assessor found it difficult to rate targets 1–7 in this case. It turned out that in this consultation little MI was used. In another short MI-part (2.5 min) the assessors had no difficulties to rate the targets. The difference was that MI was explicitly used in this consultation.

c. The length of the descriptions of the targets
  The descriptions of the targets of the MITS are comprehensive and divided over ‘summary’, ‘definition’ and ‘elaboration’ (see Fig. 1). The length of these descriptions however led to the undesirable effect that each assessor made its own summary of the most important aspects in order to facilitate decision-making while assessing. During the evaluation meetings shorter descriptions, in which the core components are reflected, were discussed and formulated. The short description of target 6 (Evocation) became:
*“An evocative style should be maintained throughout the interview. The practitioner assists the person to articulate arguments in favour of change and ideas about how change could be achieved, and does not attempt to overtly persuade the person. Change talk is encouraged and consolidated.”*

### Validity evidence in GP setting

- Representativeness of the MITS for the entire spectrum of MI
  The assessors agreed on the completeness of the targets of the MITS to assess all components of MI concerning the desirability to change and on the fact that the core concepts of MI are very well reflected in the targets. However, in general practice more attention should be paid to the follow-up of the consultation as discussing behaviour change in one consultation might be insufficient to achieve long-term behaviour change.
*“By not paying attention to the follow-up, the practitioner possibly diminishes the chance that the patient will actually take steps” (Assessor 3).*

b. Clarity and distinctiveness of target descriptions
  Most parts of the targets are clear and distinguishable. The assessors’ interpretation of the targets was discussed and consensus on how to distinguish between targets was recorded. This yielded agreements to facilitate decision-making (Table 8).

**Table 8:** Examples of added scoring instructions per target

Target	Added scoring instruction
1	Rate the process, not the content to reduce overlap with other targets
2	The behaviour should be used in a functional way. Take non-verbal behaviour into account
3	Rate the quality / functionality of the reflections. Rate other techniques when used in an empathic way
4	Rate the process, not the content
5	Rate the content, not the process
6	The practitioner should reinforce change talk but not sustain talk
7	Rate the process and content
8	Rate the process and content
9	Bad timing of structured brief tactics should be scored at target 1 and 7
10	Not giving information or advice might be seen as a shortcoming in certain consultations and can be rated here

### Distribution of the ratings

When rated with the original score options (ranging from 0 to 4), from the mandatory targets (target 1–7), target 3 (empathy) and target 5 (independence) had ratings from 1 to 4 and were not distributed over the full range of the five-point scale, while the other targets were. When rated with the adapted score options, target 3 (empathy) and target 7 (navigation) had ratings from 1 to 4. All other ratings were distributed over all five score options.

From the optional targets, only target 8 (contrasts) was not distributed over all score options but only over 2 and 3 (in the original score options) and over 1 and 2 in the adjusted score options. This optional target was rated in only five of the 27 assessments.

## Discussion

In this qualitative study, the MITS turned out to be a feasible instrument to rate MI in GP consultations. Additionally, in terms of the content validity, one of the sources of validity according to the Unified Model of Validity (Table 1), the MITS-targets adequately cover the central aspects of MI. The second source of the model, the response process validity, yielded information on how to understand and reduce response error. Suggestions will be made on how feasibility and validity can be further improved.

### Feasibility

Consultations of GPs are brief and behaviour change has to be discussed among other topics. Nevertheless we found that in all but one consultation all mandatory targets could be scored, even when little time was spent on behaviour change. Only if during this short time spent on behaviour change, little MI was used, not all targets could be scored.

Assessing more consultations (30–45 min) at once and giving one holistic impression for every target, which was the original intention of the developers, seems difficult. Even when the assessors assessed only one consultation they needed to check their notes or watch the recording again in order to score the targets. For summative assessment and research purposes we would recommend scoring consultations separately and calculate the average. This will probably contribute to the inter-rater reliability (as part of the internal structure validity) and is consistent with the use of other communication assessment tools in post-graduate education [16, 24] .

Initially, the assessors needed a considerable amount of time to assess the consultations. Familiarity with the MITS reduced the scoring time substantially. The suggestions provided in this study about unequivocal scoring instructions and shorter descriptions of the targets might facilitate decision-making as well. The duration of the assessment became within the range of other reports on scoring duration [25]. Although the assessors preferred to use a transcript, it was feasible to assess consultations without transcript. To produce transcripts is time-consuming but might partly compensate for the time spent on making notes and watching (parts of) the consultations twice.

MITS users are supposed to have a thorough understanding of MI. This seems inherent to global assessment. Ilgen et al. [24] noted that the accuracy of global assessments might be dependent upon rater-characteristics such as familiarity with the scale, clinical expertise and training whereas for checklists less expertise is required.

### Validity evidence

In this studied we analysed themes, wording and format of items of the MITS and the actions and thought processes of the assessors (including scoring) according to the Unified Model of Validity.

Burt et al. [16] noticed that global rating was preferred over checklists as it may better capture nuanced elements of expertise or deviation from desired practice. Global rating and the weighing of “nuances and deviations” is one of the strengths of the MITS and is difficult to achieve with checklists or with counting communication techniques.

Another strength of the MITS is that the rating is not constricted to a certain part of the consultation; as the assessors did not know when the change topic would be discussed, they watched the whole consultations. This generated important information. GPs frequently behaved differently when they consciously applied MI. For instance, they supported patients’ autonomy in only that part of the consultation when they where talking about “lifestyle change” but not in other parts where it could have been done as well. In the MITS, these inconsistencies can be reflected in the ratings. There are probably several reasons for the fact that GPs did not integrate the MI-skills in parts of the consultations where they could have been used as well. Often, GPs learn MI during educational programs and from guidelines or books that address smoking cessation. As a result, they might be less focused on the use of MI in other contexts. They also might find it difficult to use MI in other situations or they just don’t know the broad range of health behaviour in which MI can be used. The fact that most consultations in this study were also on smoking cigarettes might support this assumption.

The targets of the MITS consist of a variety of descriptors; summaries, definitions, and elaborations (Fig. 1). Some of these descriptions might provide a slightly different emphasis. This forces the assessor to choose what is the most appropriate in that situation. To avoid that MITS-users create their own ‘summaries’ of the targets, we would recommend using short target descriptions during scoring. This will contribute to response process validity. The same problem applies to the variety of adverbs and synonyms used in the target score options. The original score options contain a mixture of quantitative and qualitative adverbs, we used only four qualitative adverbs in the adjusted score options and added the quantity per score option in order to make them less ambiguous.

The authors chose to restrict the targets to observable MI behaviour. However, we added ‘missed opportunities’, ‘wrongly unused’ behaviour and ‘MI-inconsistency’ to the score options as the assessors felt that they otherwise could not assess the quality of MI in a proper way. The addition of ‘MI-inconsistency’ is in line with earlier research findings [6, 9] that avoidance of MI-inconsistent skills is related to better outcome. Also, from an educational perspective it is desirable that these concepts are explicitly stated so that feedback can be given. The added concepts contribute to the response process validity.

Although the MITS covers the core aspects of MI, in the GP setting we would recommend to add ‘discussion of the follow-up’ to target 9. Examples include: asking permission to discuss the behaviour change in the future, refer the patient to another care provider or discussion of the implementation of the intended steps.

### Limitations

Several aspects possibly reduced the diversity of the consultations. First, although MI can be used for all kinds of behavioural change most consultations were about smoking cessation: in seven out of nine consultations this was the case. Although this could have affected the range of skills used during the consultations in general, we do not think that this applies to the MI-skills. Moreover, the MITS is a global intrument and the targets are suitable to measure MI for all kinds of health behaviour.

Second, even though we emphasized that any consult addressing behavioural change could be handed in by the participants, irrespective of the quality of MI performed, it might have been that participants were inclined to hand in their ‘best consultation’. If this were the case, this would have reduced the range of MI-proficiency. Third, all data were derived from one institute, which might also have been at the expense of the diversity. However, despite these possible restrictions with respect to the diversity of the consultations being assessed, the data showed that for almost all targets all answering options were being used.

An additional limitation is that we changed the procedure and assessed every consultation instead of giving our impression of all consultations at once. This may have been at the expense of the holistic impression but matches the way in which communication assessment tools are used in GP-training and facilitates giving feedback to trainees.

## Conclusion

The MITS is a carefully constructed instrument representing the broad spectrum and current understanding of MI and its global nature is in line with the view on how to assess complex communication skills in brief consultations, in which providers have only short time to apply MI. With some adjustments, the MITS seems a valuable tool to assess the quality of MI in general practice.

Now that we found evidence for content and response process validity, further research should focus on the other sources mentioned in the Unified Model of Validity, namely: ‘internal structure evidence’ (reliability and factor structure), ‘relations to other variables’ (correlation with other instruments) and evaluation of intended and unintended ‘consequences’ of assessment with the MITS.

## Additional file

Additional file 1: Semi-gestructureerde vragenlijst beoordeling MITS: This is the Dutch questionnaire that the assessors filled out when assessing the consultations. (DOCX 16 kb)

## Abbreviations

BECCI: Behaviour change counselling index
MI: Motivational interviewing
MISC: Manual for the motivational interviewing skill code
MITI: Motivational interviewing treatment integrity
MITS: Motivational interviewing target scheme

## Footnotes

1 From here on GP will refer to GPs and GP-trainees
