# Supplementary material for: Assessment of motivational interviewing: a qualitative study of response process validity, content validity and feasibility of the motivational interviewing target scheme (MITS) in general practice
Source: BMC Med Educ. 2017 Nov 21;17:224. doi: 10.1186/s12909-017-1052-7 (PMC5698949; doi:10.1186/s12909-017-1052-7)
Supplement: Additional file 1: — Semi-gestructureerde vragenlijst beoordeling MITS: This is the Dutch questionnaire that the assessors filled out when assessing the consultations. (DOCX 16 kb) [file 12909_2017_1052_MOESM1_ESM.docx]

**Additional file 1 Semi-gestructureerde vragenlijst beoordeling MITS**

Naam consult:

Naam beoordelaar:

Neem de tijd op die je besteed aan het beoordelen.

**Hoe ga je te werk:**

1x consult luisteren/bekijken (en/of meelezen met verbatim). Je mag het gesprek wel tussendoor stil zetten.

1. Doelgedrag scoren (0-4) en invullen in schema

| Doelgedrag Target | Score originele ss en opmerkingen | Score aangepaste ss en opmerkingen |
| --- | --- | --- |
| 1 |  |  |
| 2 |  |  |
| 3 |  |  |
| 4 |  |  |
| 5 |  |  |
| 6 |  |  |
| 7 |  |  |
| 8 |  |  |
| 9 |  |  |
| 10 |  |  |

2. Welke problemen kwam je tegen?

Wat was moeilijk? En waar ligt dat aan?

Wat was gemakkelijk? En waar ligt dat aan?

3. Als je het idee hebt dat je niet genoeg hebt aan 1x luisteren om de volgende vraag te beantwoorden, luister dan nog een keer voordat je de volgende vraag beantwoord.

Heb je nog een keer geluisterd? Ja / nee

4. Is het consult geschikt voor het onderzoek? Zo nee waarom niet? Zo ja waarom wel?

Zo ja welke onderdelen zijn niet/wel van belang?

5. Voor welke (lichamelijke) klachten komt deze patiënt bij de huisarts?

-

-

6. Welke gedragsverandering wordt besproken?

-

-

7. Hoe lang duurde het consult?...................min

8. Hoe groot is het deel van het consult dat over MI gaat ongeveer**?** .........min

9. Hoe lang ben je in totaal bezig geweest met beoordelen ? ………min
